# Mechanism of Resistance and Novel Targets Mediating Resistance to EGFR and c-Met Tyrosine Kinase Inhibitors in Non-Small Cell Lung Cancer

**DOI:** 10.1371/journal.pone.0136155

**Published:** 2015-08-24

**Authors:** Gregory M. Botting, Ichwaku Rastogi, Gagan Chhabra, Marie Nlend, Neelu Puri

**Affiliations:** 1 Department of Biomedical Sciences, University of Illinois College of Medicine, Rockford, Illinois, United States of America; 2 Thermo Fisher Scientific, Rockford, Illinois, United States of America; H. Lee Moffitt Cancer Center & Research Institute, UNITED STATES

## Abstract

Tyrosine kinase inhibitors (TKIs) against EGFR and c-Met are initially effective when administered individually or in combination to non-small cell lung cancer (NSCLC) patients. However, the overall efficacies of TKIs are limited due to the development of drug resistance. Therefore, it is important to elucidate mechanisms of EGFR and c-Met TKI resistance in order to develop more effective therapies. Model NSCLC cell lines H1975 and H2170 were used to study the similarities and differences in mechanisms of EGFR/c-Met TKI resistance. H1975 cells are positive for the T790M EGFR mutation, which confers resistance to current EGFR TKI therapies, while H2170 cells are EGFR wild-type. Previously, H2170 cells were made resistant to the EGFR TKI erlotinib and the c-Met TKI SU11274 by exposure to progressively increasing concentrations of TKIs. In H2170 and H1975 TKI-resistant cells, key Wnt and mTOR proteins were found to be differentially modulated. Wnt signaling transducer, active β-catenin was upregulated in TKI-resistant H2170 cells when compared to parental cells. GATA-6, a transcriptional activator of Wnt, was also found to be upregulated in resistant H2170 cells. In H2170 erlotinib resistant cells, upregulation of inactive GSK3β (p-GSK3β) was observed, indicating activation of Wnt and mTOR pathways which are otherwise inhibited by its active form. However, in H1975 cells, Wnt modulators such as active β-catenin, GATA-6 and p-GSK3β were downregulated. Additional results from MTT cell viability assays demonstrated that H1975 cell proliferation was not significantly decreased after Wnt inhibition by XAV939, but combination treatment with everolimus (mTOR inhibitor) and erlotinib resulted in synergistic cell growth inhibition. Thus, in H2170 cells and H1975 cells, simultaneous inhibition of key Wnt or mTOR pathway proteins in addition to EGFR and c-Met may be a promising strategy for overcoming EGFR and c-Met TKI resistance in NSCLC patients.

## Introduction

EGFR and c-Met are receptor tyrosine kinases (RTKs) that are highly expressed in NSCLC and facilitate tumorigenic signaling through shared pathways when dysregulated [1,2]. Several tyrosine kinase inhibitor (TKI) therapies against EGFR and c-Met are currently administered and are initially effective in NSCLC patients who have certain somatic EGFR-activating mutations such as L858R [3–5]. However, the development of TKI resistance is common and results in the recurrence of tumors [6,7]. Greater than 50% of all acquired secondary resistance to EGFR TKIs is attributed to the development of the T790M secondary ‘gatekeeper mutation’ [8–12]. This mutation may also cause primary EGFR TKI resistance if present prior to treatment [10]. Another 20% of acquired resistance to EGFR TKIs is attributed to amplification of the c-Met receptor [2,13,14]. *MET* gene amplification and the presence of T790M are not mutually exclusive, as studies have shown that many NSCLC patients are positive for both alterations [2,15].

Previous studies by our group and others have demonstrated that EGFR and c-Met have substantial cross-talk which contributes to increased activation of their shared downstream pathways [16]. Also evidence has been provided that there is a synergistic effect between EGF and HGF on tumorigenicity [1], and that EGFR and c-Met TKIs can synergistically inhibit NSCLC cell proliferation [17].

Research has suggested that dysregulation of the Wnt pathway may be an important factor contributing to enhanced maintenance and proliferation signaling in various cancers [18,19]. Other studies suggest that crosstalk between EGFR and Wnt may enhance lung cancer tumorigenesis [17,18,20]. XAV939, a tankyrase inhibitor is a promising small-molecule Wnt inhibitor currently in preclinical studies. XAV939 activates Axin1, promoting β-catenin degradation [21], and thus inhibition of canonical Wnt signaling. Furthermore, Mammalian target of rapamycin (mTOR), a serine/threonine kinase which is a key player in the PI3K/Akt pathway, acting both up and downstream of Akt [22–25] has also been linked with a variety of cancers when dysregulated. Thus, mTOR has also become a potential therapeutic target in anti-cancer therapies [26]. Rapamycin and its derivative, everolimus, are two promising mTOR inhibitors currently in clinical trials for lung cancer [27–30]. Canonical Wnt and mTOR pathways can be negatively regulated by the serine/threonine kinase GSK3β [31–33]. In humans, GSK3 has two isoforms, GSK3α and GSK3β [34], with the latter being known to function as part of the β-catenin destruction complex[33,35,36]. This investigation compares these alternative signaling pathways, specifically key proteins of the Wnt and mTOR pathways, in model NSCLC cell lines positive or negative for EGFR-activating mutation T790M.

Recent studies in our laboratory involving TKI-resistant H2170 cells have demonstrated an upregulation of p-ERK, a protein which is known to activate GATA-6 [17]. GATA-6 is a transcription factor believed to be essential for the development of lung epithelial cells and other embryogenic processes [37,38], by regulating the Wnt pathway [37]. GATA-6 is also known to facilitate Wnt activation by promoting the transcription of important Wnt ligands [37,39–43]. Stimulation of the canonical Wnt pathway ultimately results in the activation of β-catenin (dephosphorylated on Ser37 and Thr41), which promotes the transcription of proteins involved in cell proliferation [44,45].

This study demonstrates that combining Wnt or mTOR inhibitors with current EGFR and c-Met TKIs may successfully inhibit cell proliferation and survival in wild-type EGFR NSCLC cells. However, in the case of T790M-positive cells, it may be possible to break resistance, through combining mTOR inhibitors with EGFR and c-Met TKIs. This study suggests that the mechanism of resistance to EGFR/c-Met TKI’s is different in mutated (T790M) and wild type EGFR NSCLC cells. This indicates that selective combinatorial treatment should be used for cells with wild type EGFR and T790M mutated EGFR, to improve lung cancer patient prognosis.

## Materials and Methods

### Reagents and Antibodies

Erlotinib [*N*-(3-ethynylphenyl)-6,7-bis(2-methoxyethoxy) quinazolin-4-amine] (Cat. No. E-4007) and everolimus (Cat. No. E-4040) were purchased from LC Laboratories (Woburn, MA), SU11274 [[(3Z)-N-(3-chlorophenyl)-3-({3,5-dimethyl-4-[(4-methylpiperazin-1-yl) carbonyl]1H-pyrrol-2-yl}methylene)-N-methyl-2-oxo-2,3-dihydro-1H-indole-5-sulfonamide]] (Cat. No. S-9820) and XAV939 [3,5,7,8-tetrahydro-2-[4-(trifluoromethyl)phenyl]-4H-thiopyrano[4,3-d]pyrimidin-4-one] (Cat. No. 53113) were purchased from Sigma-Aldrich (St. Louis, MO). All inhibitors were suspended in DMSO and stored as aliquots at -20°C. EGF (Cat. No. AF-100-15) and HGF (Cat. No. 100–39) were purchased from PeproTech (Rocky Hill, NJ) and were suspended in PBS and stored as aliquots at -20°C.

Phosphospecific rabbit monoclonal antibodies for p-mTOR (Ser 2448, Clone D9C2), p-4E-BP1 (Thr37/46, Clone 2855), p-GSK3β (Ser 9), Total GSK3β, Axin1 (C76H11) and p-LRP6 (C5C7), phosphospecific rabbit polyclonal antibodies for p-ERK1/2 (Thr202/Tyr204) and p-p70SK (T389), rabbit and mouse IgG secondary antibodies were obtained from Cell Signaling Technology. Rabbit polyclonal unphosphorylated GATA-6 (sc-9055) was obtained from Santa Cruz Biotechnology (Santa Cruz, CA). A mouse monoclonal antibody for active β-catenin (05–665) was obtained from Millipore (Billerica, MA). A mouse monoclonal antibody for β-actin was obtained from Sigma-Aldrich (St. Louis, MO). All antibodies were used according to the manufacturer’s instructions.

### Cell Lines and Cell Culture

H2170 and H1975 NSCLC cell lines were purchased from American Type Culture Collection (ATCC) (Rockville, MD, USA, CRL-5928, CRL-5807 and CRL-5908, respectively). The H2170 cells have wild-type EGFR, while H1975 cells are positive for two EGFR kinase domain mutations: L858R and T790M (documented by ATCC). All cell lines were stored in incubators at 37°C with 7% CO_2_ and were cultured according to ATCC instructions (atcc.org) in Roswell Park Memorial Institute (RPMI 1640) media (Thermo Fisher Scientific, Pittsburg, PA, Cat No: SH3002701) supplemented with 10% (v/v) Fetal Bovine Serum (Atlanta Biologicals, Lawrenceville, GA, Cat No: S11050), 1% (v/v) Antibiotic-Antimycotic Solution (Life Technologies, Carlsbad, CA, Cat No: 15070–063), 1% (v/v) Sodium Pyruvate (Life Technologies, Carlsbad, CA, Cat No: 11360) and 1% (v/v) Hepes (Life Technologies, Carlsbad, CA, Cat No: 11360).

### Effect of EGF/HGF and TKIs on Phosphorylation of EGFR, c-Met and Other Signaling Pathways

Cells were treated before lysis for determining the effects of growth factor ligands and TKIs on protein expression. Parental cells were plated and allowed to adhere and grow for 24 to 48 hours until dishes were approximately 40% confluent. Cells were then starved for 24 hours with serum-free RPMI (with 0.5% BSA). After 24 hours of starvation, cells were treated with or without respective TKIs (erlotinib or SU11274) for 24 hours. After 24 hours of TKI treatment, cells were treated with or without growth factor ligands (15 ng/mL EGF for 2.5 minutes or 40 ng/mL HGF for 7.5 minutes at 37°C). Immediately following ligand treatment, cells were lysed and collected for immunoblotting.

### Cell Lysis and Immunoblotting

Following all cell pre-treatments (as described above), cells were lysed in buffer (20 mM Tris, 150 mM NaCl, 10% glycerol, 1% NP-40, 0.42% NaF, 1 mM phenylmethylsulfonyl fluoride, 1 nM sodium orthovanidate, and 10 mM protease inhibitor cocktail (Sigma-Aldrich). Cell lysates were then electrophoresed for separation on 7.5% or 10% SDS-PAGE. Separated proteins were then transferred on to nitrocellulose membranes (Bio-Rad Laboatories, Hercules, CA) and probed with antibodies for Wnt or mTOR pathway related proteins. Immunoblots were developed using Pierce ECL Substrate chemiluminescence kit (Thermo Fisher Scientific, Rockford, IL, 32109) and modulations of different proteins were calculated by densitometric analysis using NIH ImageJ software.

### MTT Cell Viability Assay

The effects of various TKIs on H2170 and H1975 cell viability were measured by MTT colorimetric dye reduction assay using MTT 3-(4,5-dimethylthiazol-2-yl)-2,5-diphenyltetrazolium bromide dye according to the manufacturer’s instructions. Cells were plated at 3000 cells per well on 96-well plates and after 24 hours, cells were treated with inhibitors for 72 hours. MTT reagent (Sigma-Aldrich, St. Louis, MO, Cat. No. M5655) was then added, and cells were incubated at 37°C for 4 hours, after which formazan crystals were solubilized and absorbance was measured at a wavelength of 570 nm. Percentage viability of drug treated cells was calculated relative to untreated control cells. All cell viability experiments were performed three times in replicates of six for each treatment condition.

### Immunofluorescence

20,000 cells were plated in 8-well glass chamber slides per well (Lab-Tek II Chamber Slide System, Thermo Fisher Scientific, Rockford, IL) which were pre-coated with poly-L-lysine (0.01% solution) (Sigma-Aldrich, St. Louis, MO). Cells were allowed to adhere for 24 hours, after which cells were starved overnight in serum-free medium (with 0.5% BSA). Cells were then treated with EGF/HGF (15 ng/mL EGF for 2.5 minutes or 40 ng/mL HGF for 7.5 minutes at 37°C), and fixed using 4% paraformaldehyde solution in 1X PBS. Cells were then permeabilized (0.1% Triton X-100 solution in 1X PBS) and blocked (buffer containing 5% normal goat serum in 1X PBS). Cells were incubated overnight with active β-catenin primary antibody (1:400) at 4°C and then incubated for 1 hour at room temperature with IgG DyLight 488 anti-mouse secondary antibody (1:250) (Thermo Fisher Scientific, Rockford, IL) (Product #35502) diluted in 1X PBS with 1% BSA and Hoechst nuclear staining dye (blue). Cells were observed using a Zeiss Axio Observer Z1 microscope. Average nuclear fluorescence intensity of active β-catenin staining was measured using NIH ImageJ software over 10 microscopic fields per treatment condition and values were averaged.

### qPCR analysis

200,000 cells were plated in a 35 mm dish and were allowed to adhere for 24 hours. Starving media (RPMI with 0.5% BSA) was then added to the cells for 24 hours, after which total RNA was collected using Invitrogen RNA mini kit. The RNA was quantified using Take 3 nanodrop and then equal concentrations of RNA were used for synthesis of cDNA. The primer sequences used for β-Catenin are F: TGGATGGGCTGCCTCCAGGTGAC and R: ACCAGCCCACCCCTCGAGCCC and for GAPDH are F: TTGCCAATGACCCCTTCA and R: CGCCCCACTTGATTTTGGA. qPCR was performed using SuperScript III Platinum Two-Step qRT-PCR Kit (Life Technologies, Carlsbad, CA) according to manufacturer’s protocol. The expression of each gene was analyzed in triplicates and the Ct values were normalized with GAPDH. The data was then analyzed using ΔΔCt method and fold changes were calculated using 2^(-ΔΔCt)^.

### Statistical analysis

All the experiments were performed three to five times. The Student’s t-test or ANOVA was used to analyze the statistical significance of the data. A p-value of less than 0.05 was considered to be statistically significant throughout the study.

## Results

### Comparison of IC_50_ of parental and resistant H2170 cell lines with H1975 cell line

H2170 cells were initially moderately sensitive to TKIs erlotinib and SU11274 due to their EGFR wild-type status (Table 1). As described in our earlier study [17], parental (naïve) H2170 cells were treated with increasing concentrations of erlotinib (0.5 to 14 μM) and SU11274 (2.5 to 17 μM) over several months to obtain cells with stable resistance at high concentrations of these TKIs. These cells exhibited stable resistance after 12 passages in drug free media. MTT cell viability assays were performed for determining the IC_50_ for erlotinib and SU11274. The IC_50_ values were calculated using Sigma Plot 12.5 software and results are displayed in Table 1. The IC_50_ of erlotinib and SU11274 in H2170 erlotinib resistant (H2170-ER) and SU11274 resistant (H2170-SR) cells was found to be 11 to 22-fold and 4 to 5-fold greater respectively [17], when compared to H2170 parental (H2170-P) cells. However, the IC_50_ of erlotinib and SU11274 were found to be approximately 15-fold and 2-fold higher, respectively, in H1975 cells, when compared to H2170 parental cells. During this study, H2170 TKI-resistant cells were maintained in media containing 10 μM erlotinib or 10 μM SU11274. H1975 cells are naturally resistant to erlotinib, due to the presence of the T790M mutation, and for the purpose of this study H1975 cells were cultured in drug-free medium.

**Table 1:** IC_50_ of RTKIs for NSCLC cell lines with and without T790M mutation.

Cell line	Erlotinib	SU11724
H2170 Parental ^[^ ^17^ ^]^	0.5μM	2.5μM
H2170 Resistant ^[^ ^17^ ^]^	11μM	12μM
H1975	7.62μM	4.74μM

### Dual inhibition by EGFR and c-Met TKIs on H1975 cell proliferation

**Fig 1:**
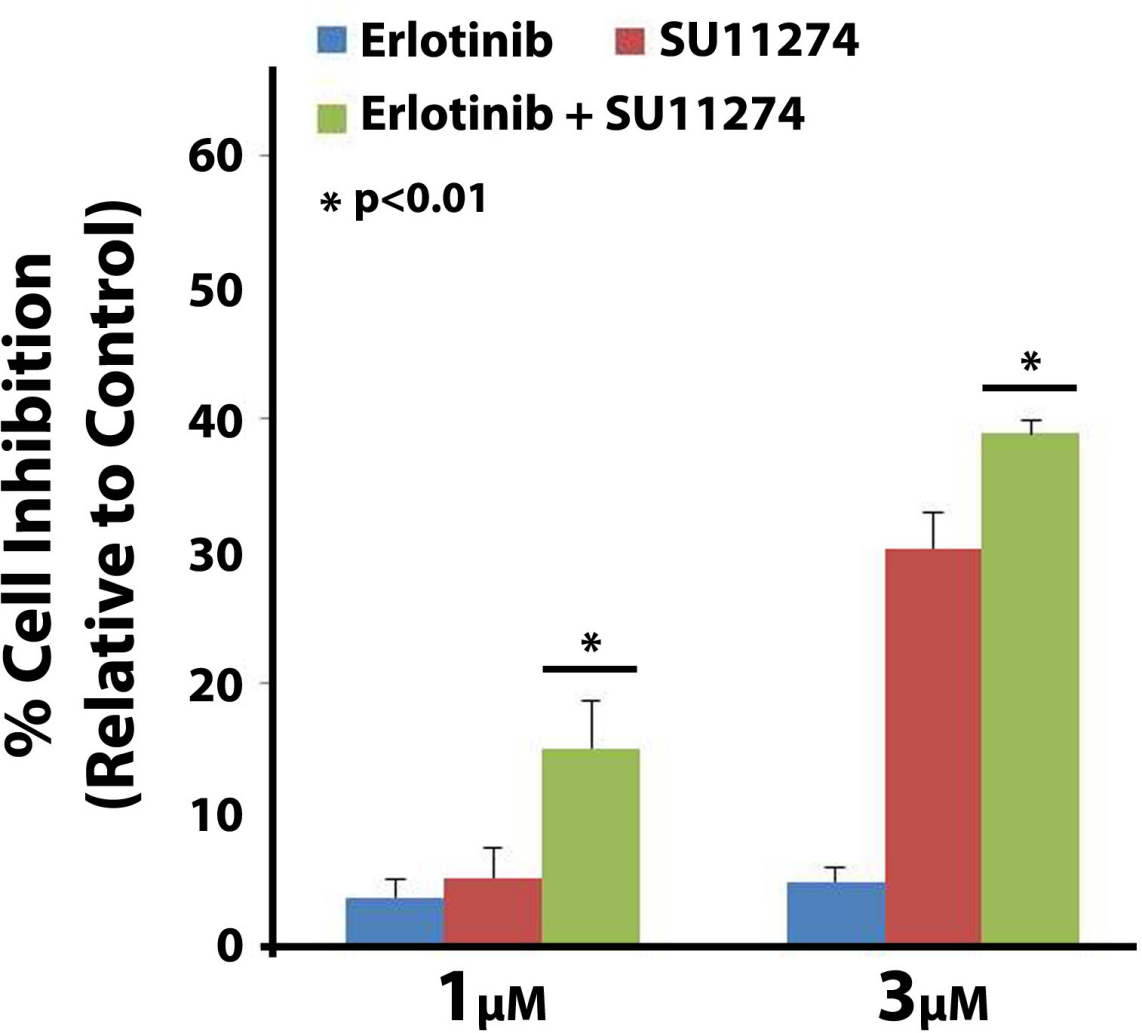
Erlotinib and SU11724 synergism on H1975 cells. H1975 cells were plated at 3000 cells per well in a 96 well plate and after 24 hours were treated with varying combinations of EGFR inhibitor erlotinib and c-Met inhibitor SU11274. After 72 hours of drug exposure MTT cell viability assay was performed. Synergistic inhibitory effects on H1975 cell growth inhibition were observed following combination treatment of erlotinib and SU11274 at concentrations of 1 μM and 3 μM (1:1 ratio of each drug). Drug synergism was calculated using Calcusyn v2.0 software and the CI values were below 1. ANOVA analysis was used to determine statistically significant differences between treatments (n = 3, p<0.01).

Since, the T790M EGFR mutation is known to confer erlotinib resistance (Table 1) in NSCLC, it is important to identify potential drug susceptibility caused by this mutation. Therefore, we tested for drug synergism on H1975 cells (positive for the L858R and T790M EGFR mutations) using erlotinib and SU11274 via MTT cell viability assay. Synergistic effects on H1975 cell growth inhibition were observed with erlotinib and SU11274 in combination at concentrations of 1 μM (1:1 ratio of each drug) and 3 μM (1:1 ratio of each drug) (Fig 1). However, their combinatorial effects were not synergistic above the concentration of 5 μM of each drug (1:1 ratio) (data not shown). Synergism was determined using Calcusyn software v2.0 and combinatorial index (CI) values below 1 were obtained (CI values <1 indicate synergism) [46].

### Role of Wnt and mtor pathways in EGFR/c-Met TKI-resistance

**Fig 2:**
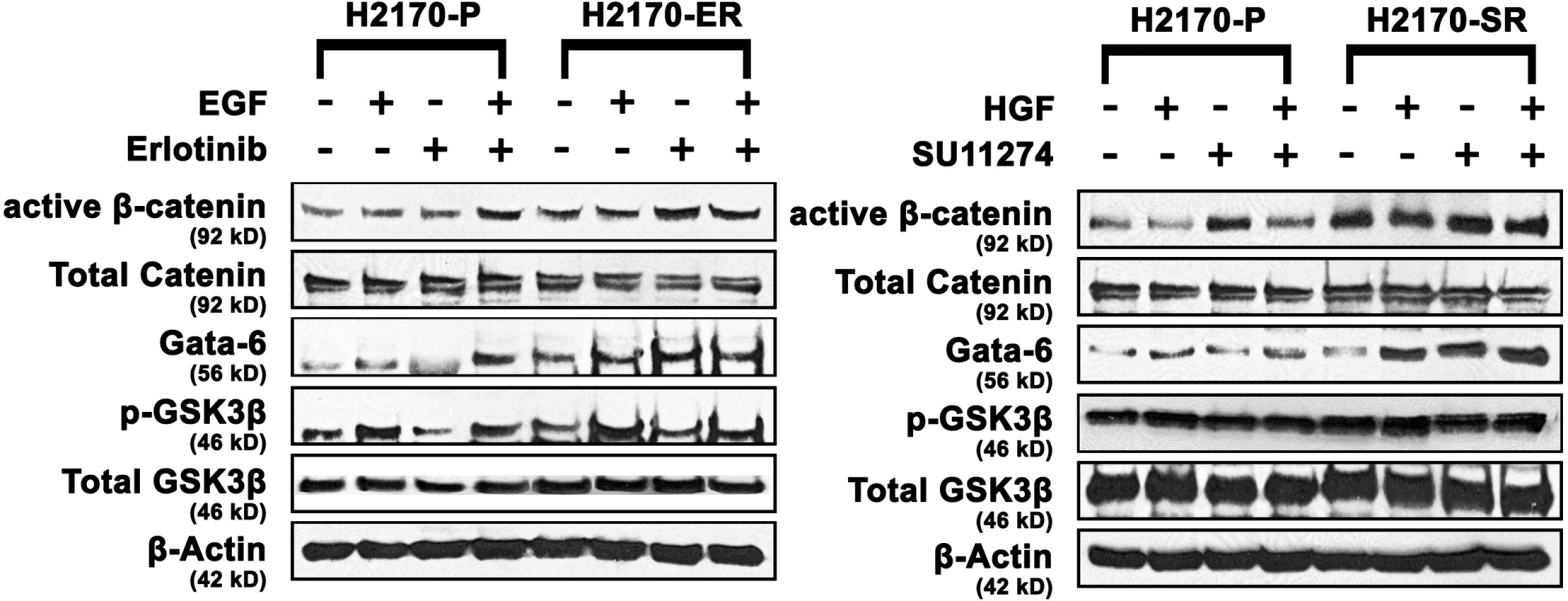
Modulation of key Wnt and mTOR proteins in H2170 TKI-resistant cells. H2170-P, H2170-ER and H2170-SR cells were plated in 35 mm dishes at 125,000 cells per dish and starved (RPMI 1640 with 0.5% BSA) for 24 hours before ligand (EGF and HGF) or/and drug (erlotinib and SU11274) treatments. After western blot analysis increased expression of Wnt and mTOR pathway related proteins in H2170-ER and H2170-SR cells were observed when compared to H2170-P cells with the exception of p-GSK-3β in H2170 SR cells. The fold changes were calculated using ImageJ software. (n = 3, p<0.05).

In order to elucidate mechanism of resistance to erlotinib and SU11274 in H2170 cells, expression levels of key proteins involved in Wnt/mTOR pathway were determined by immunoblotting. We observed that active β-catenin was upregulated 1.5-fold and 2.0-fold in the presence of EGF and erlotinib respectively, in H2170-ER cells, when compared to same treatments in H2170 parental cells. We also observed that GATA-6 was upregulated 2.0 to 3.0-fold in the presence and absence of EGF and erlotinib in H2170-ER cells when compared to same treatments in H2170-P cells. Furthermore, p-GSK3β (Ser9) was found to be upregulated approximately 2-fold in the H2170-ER cells in the presence of erlotinib when compared to the erlotinib treated H2170-P cells (Fig 2).

Similarly, in the H2170-SR cells, we observed active β-catenin was upregulated 2.0 to 4.0-fold in the presence and absence of HGF and SU11274; p-GSK-3b was upregulated 1.5 to 2.0 fold in the presence and absence of HGF and SU11274; and GATA-6 was also upregulated 3.0 to 4.0-fold in the presence of HGF and SU11274, when compared to same treatments in H2170-P cells (p<0.01) (Fig 2). No significant modulation in the expression of total proteins was observed among the H2170-P, H2170-ER and H2170-SR cells (Fig 2).

### Increased nuclear accumulation of active β-catenin in H2170-ER and H2170-SR cells

**Fig 3:**
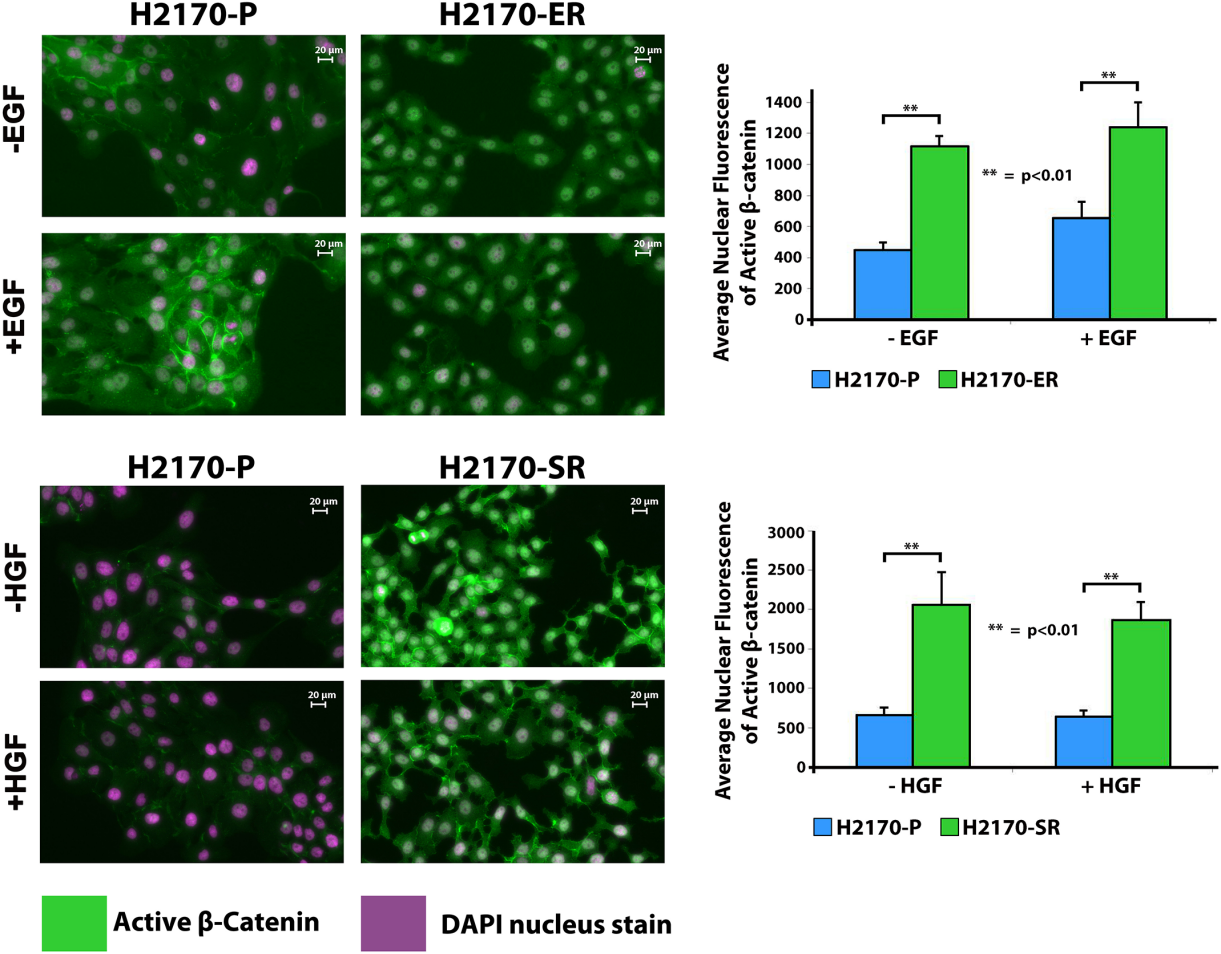
Enhanced nuclear accumulation of active β-catenin in TKI-resistant H2170 cells. H2170-P, H2170-ER and H2170-SR cells were plated in 8-well chamber slides at 20,000 cells per well and then starved overnight. Cells were fixed with 4% paraformaldehyde, permeabilized with 0.1% Triton X-100 and then blocked with 5% Normal goat serum and 0.3% Triton X-100 before incubation with primary antibody. The cells were then incubated with Dylight 488 conjugated secondary antibody and then were observed under the fluorescence microscope. The green color in the image represents active β-Catenin staining in the cell and the purple color represents DAPI nuclear staining. Average intensity of active β-catenin staining was quantitatively measured using ImageJ software. We observed greater nuclear accumulation of active β-catenin in H2170-ER and H2170-SR cells, as compared to H2170-P cells (n = 3, p<0.01).

Due to the fact that active β-catenin is a key downstream effector in the canonical Wnt signaling pathway and is observed to be significantly upregulated in H2170-ER and H2170-SR cells (Fig 2). We analyzed the localization of active β-catenin in H2170-P, H2170-ER and H2170-SR cells using immunofluorescence. Upon activation, β-catenin accumulates into the nucleus, and interacts with TCF/LEF to initiate transcription. We observed that in H2170-ER and H2170-SR cells, there was increased localization of β-catenin in nucleus when compared to H2170-P cells. Average fluorescent intensity of active β-catenin staining in the nucleus was found to be 1.9 and 2.5-fold greater in H2170-ER cells compared to H2170-P, in EGF treated and untreated cells, respectively (p<0.01) (Fig 3). Similarly, the average fluorescence intensity of active β-catenin staining in the nucleus was found to be 2.9 and 3.1-fold greater in H2170-SR cells, compared to H2170-P cells, in HGF treated and untreated cells, respectively (p<0.01) (Fig 3).

### Activation of the mtor pathway in H1975 cells

**Fig 4:**
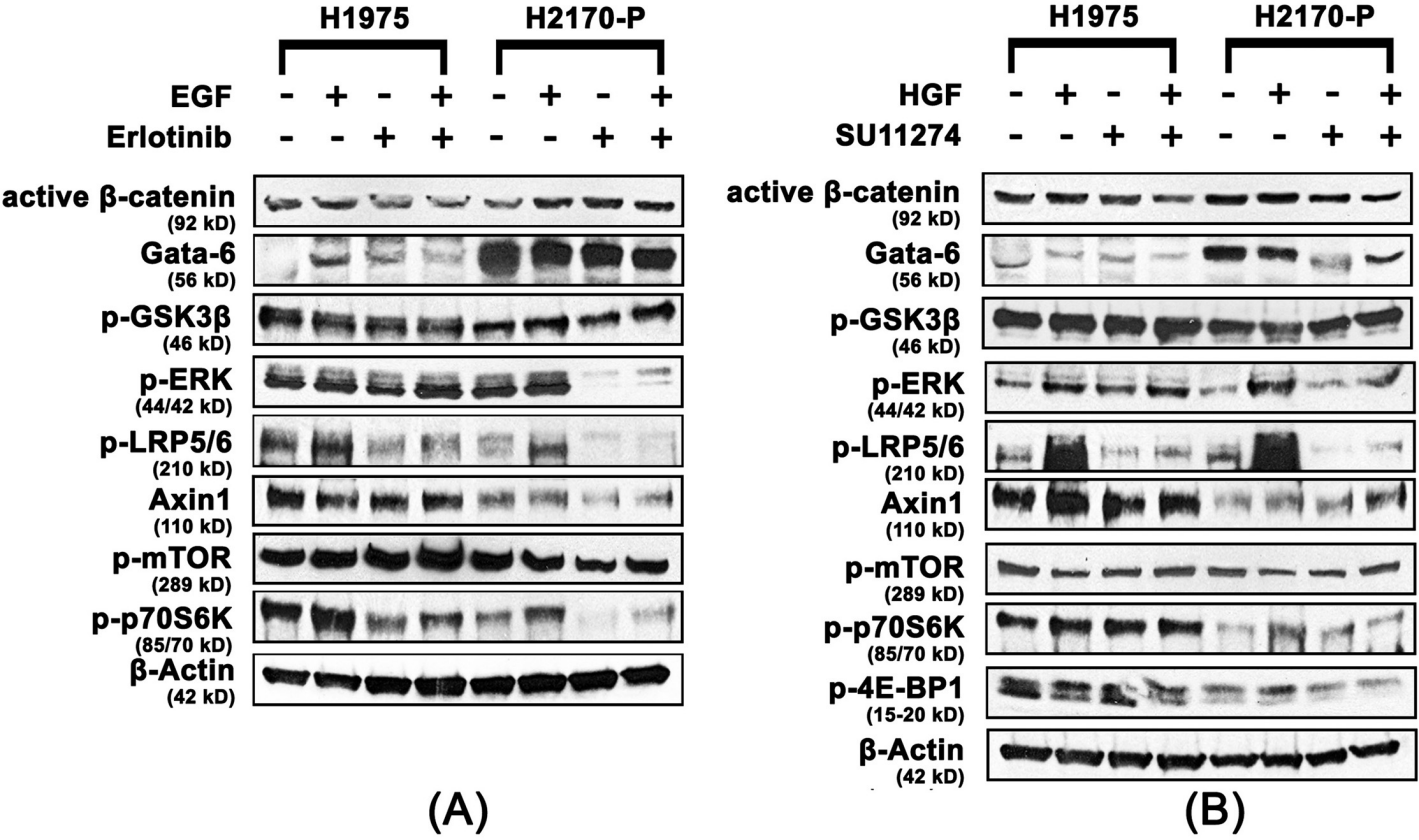
Upregulation of the mTOR pathway in H1975 cells. H1975 and H2170-P cells were plated at 125,000 cells per dish in 35 mm dishes and starved (RPMI 1640 with 0.5% BSA) for 24 hours before ligand (EGF and HGF) or/and drug (erlotinib and SU11274) treatments and analyzed using western blot. (A) Active β-catenin and GATA-6 were observed to be downregulated and p-ERK, p-mTOR, p-p70S6K, p-LRP5/6 and Axin1 were observed to be upregulated in H1975 cells when compared to the same treatments in H2170-P cells (n≥3, p<0.05). (B) Similarly active β-catenin and GATA-6 were observed to be downregulated while, p-GSK3β was not significantly modulated in H1975 cells when compared with H2170-P cells with same treatments. We also observed p-ERK, p-LRP5/6, p-mTOR, p-p70S6K, p-4E-BP1 and Axin1 were all upregulated in H1975 cells when compared to H2170-P cells with same treatments (n≥3, p<0.05).

In order to elucidate the mode of resistance to erlotinib and SU11274 in the H1975 cell line (T790M-positive), immunoblotting was performed to analyze the modulations in expression levels of important Wnt and mTOR proteins. Active β-catenin was found to be downregulated 1.5-fold in H1975 cells in the presence of erlotinib and EGF when compared to H2170-P cells with same treatments. GATA-6 was found to be downregulated 1.5 to 6.5-fold in H1975 cells in the presence and absence of EGF and erlotinib treatment when compared to same treatments in H2170-P cells. Furthermore, negative Wnt regulator Axin1 was found to be upregulated 1.5-fold and 2.5-fold in H1975 cells, compared to H2170-P cells, in the presence of EGF and erlotinib, respectively (Fig 4A). The modulations in expression of key Wnt pathway related proteins, following EGF and erlotinib treatment suggest that in H1975 cells, the Wnt/β-catenin pathway is not directly involved in development of erlotinib resistance.

Additionally, p-ERK was upregulated 10.5 and 4.6-fold in H1975 cells in the presence of erlotinib and both erlotinib and EGF, respectively, compared to same treatments in H2170-P cells. Also, p-mTOR was upregulated 1.5-fold in H1975 cells when compared to H2170-P cells in the presence of erlotinib. Furthermore, p-p70S6K was found to be upregulated 1.5-fold to 3.6-fold in H1975 cells in the presence and absence of EGF and erlotinib when compared to same treatments in H2170-P cells. Interestingly, p-LRP5/6, a common Wnt co-receptor, was found to be upregulated 1.8 to 2.5-fold in H1975 cells in the presence and absence of erlotinib when compared to same treatments in H2170-P cells (p<0.05) (Fig 4A).

Further, immunoblotting was performed in order to elucidate similarities or differences in protein expression in H1975 cells after treatment with HGF and SU11274. Active β-catenin was found to be downregulated 1.5 to 2.0-fold in H1975 cells in the presence and absence of SU11274 and GATA-6 was also found to be downregulated 1.5 to 3.5-fold in the presence and absence of HGF and SU11274 in H1975 cells when compared to same treatments to H2170-P cells. We did not observe any significant modulations in expression of p-GSK3β (Ser9) in H1975 cells when compared to similar treatment groups in H2170-P cells. Furthermore, negative Wnt regulator Axin1 was found to be upregulated 2.0-fold in H1975 cells, in the presence of HGF and SU11274 when compared to H2170-P cells with same treatments (p<0.05) (Fig 4B).

Additionally, p-ERK and p-LRP5/6 were observed to be upregulated up to 2.0-fold in H1975 cells, compared to H2170-P cells in presence of SU11274. Also, p-mTOR was upregulated 1.5-fold in absence of EGF and erlotinib and in the presence of erlotinib only, while p-p70S6K was upregulated 1.5 to 3-fold in the presence and absence of HGF and SU11274 in H1975 cells when compared to same treatments in H2170-P cells (p<0.05) (Fig 4B). We did not observe any significant modulation in the expression of key total proteins in the H1975 cells when compared to H2170-P cells (S1 Fig).

### Upregulation of the mtor pathway in H1975 cells compared to erlotinib-resistant and SU11274 resistant H2170 cells

**Fig 5:**
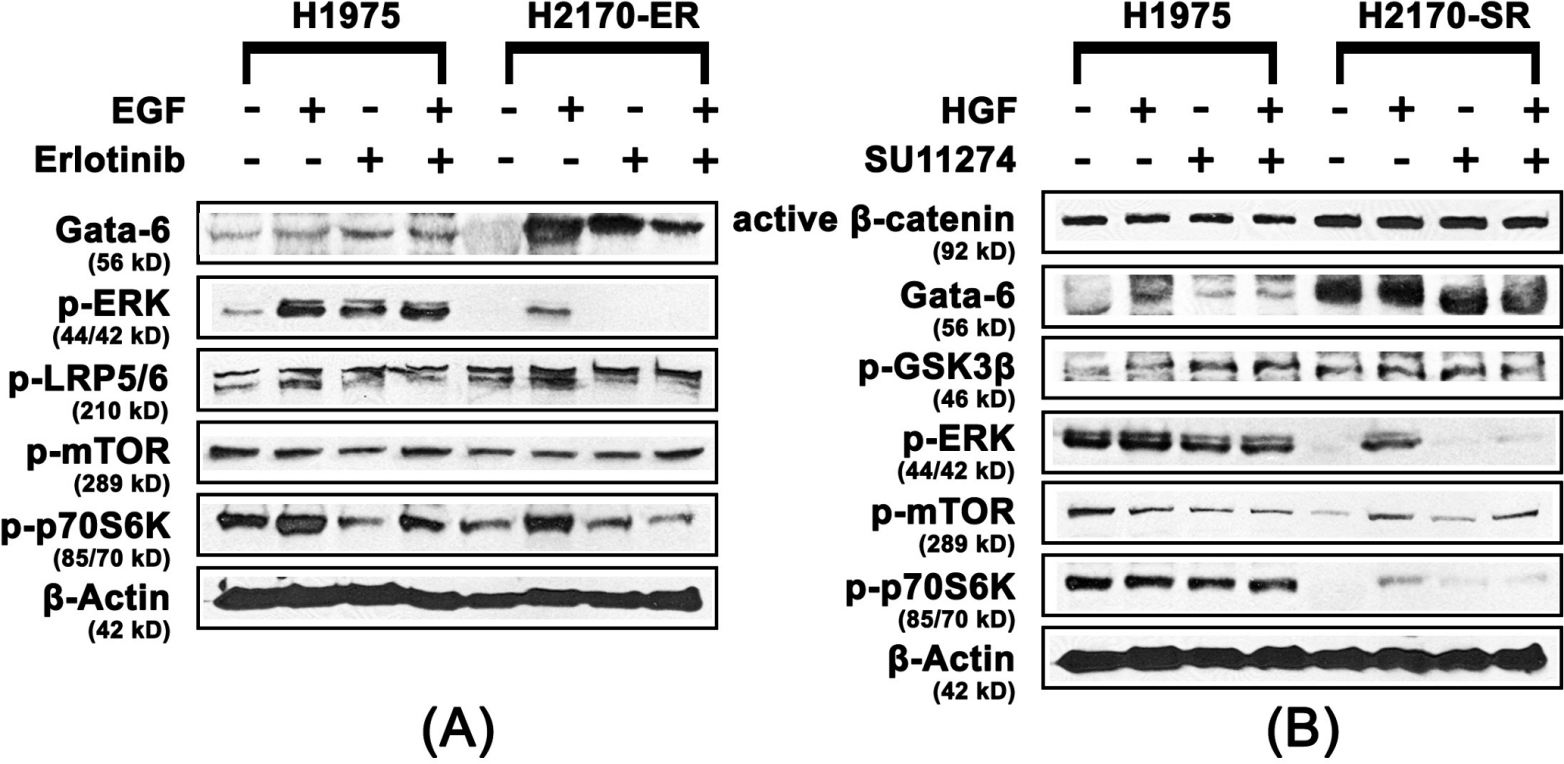
Downregulation of Wnt proteins and upregulation of mTOR proteins in H1975 EGFR-mutant cells compared H2170-ER and H2170-SR cells. H1975, H2170-ER and H2170-SR cells were plated at 125,000 cells per dish in 35 mm dishes and starved (RPMI 1640 with 0.5% BSA) for 24 hours before ligand (EGF and HGF) or/and drug (erlotinib and SU11274) treatments and were analyzed using western blot. (A) GATA-6 and p-LRP5/6 were observed to be downregulated in H1975 cells when compared to H2170 ER cells in same treatments. However, p-ERK, p-mTOR and p-p70S6K were upregulated in H1975 cells when compared to H2170-ER cells in similar treatments (n≥3, p<0.05). (B) Active β-catenin and GATA-6 were observed to be downregulated in H1975 cells compared to same treatments in H2170-SR cells. We also observed that p-ERK, p-mTOR and p-p70S6K were upregulated in H1975 cells when compared to H2170-SR cells in same treatments. The fold changes were calculated using ImageJ software (n≥3, p<0.05).

For elucidating similarities or differences in the mode of resistance to erlotinib occurring in the H1975 and H2170-ER cells, immunoblotting was performed after EGF and erlotinib treatment. p-LRP5/6 was downregulated 1.5 to 2.2-fold in H1975 cells in the presence and absence of EGF and erlotinib when compared to same treatments in H2170-ER cells. GATA-6 was found to be downregulated 3.6-fold and 2.9-fold in H1975 cells when compared to H2170-ER cells in the presence of EGF and erlotinib, respectively and p-ERK was found to be upregulated 2.0 to 70.0-fold in H1975 cells in the presence and absence of erlotinib and EGF when compared to same treatments to H2170-ER cells. Also, p-mTOR was found to be upregulated up to 1.6-fold in the presence of EGF only and in absence of both EGF and erlotinib in H1975 compared to H2170-ER cells with the same treatments. Furthermore, p-p70S6K was found to be upregulated up to 2.0-fold in H1975 cells when compared to H2170-ER cells in the absence and presence of EGF and erlotinib both (p<0.05) (Fig 5A).

Similarly, for elucidating similarities or differences in the mode of TKI resistance occurring in the H1975 and H2170-SR cells, immunoblotting was performed after HGF and SU11274 treatment. Active β-catenin was observed to be downregulated 2.0-fold in H1975 cells, compared to H2170-SR cells in the presence and absence of HGF and SU11274. GATA-6 was downregulated 1.5 to 6.0-fold in H1975 cells compared to H2170-SR cells in the presence and absence of HGF and SU11274. p-ERK was found to be upregulated 2-fold and 6.0-fold in H1975 cells, compared to H2170-SR cells, in absence of HGF and SU11274 both and in the presence of SU11274 alone, respectively. p-mTOR was upregulated 5.5-fold and 1.5-fold in absence of both HGF and SU11274; and in presence of SU11274 alone, respectively, in H1975 cells, when compared to same treatments in H2170-SR cells. Furthermore, p-p70S6K was also found to be upregulated 2.0 to 6.0-fold in H1975 cells compared to H2170-SR cells in the presence and absence of HGF and SU11274 (p<0.05) (Fig 5B).

### Increased active β-catenin expression in H2170-ER and H2170-SR cells

**Fig 6:**
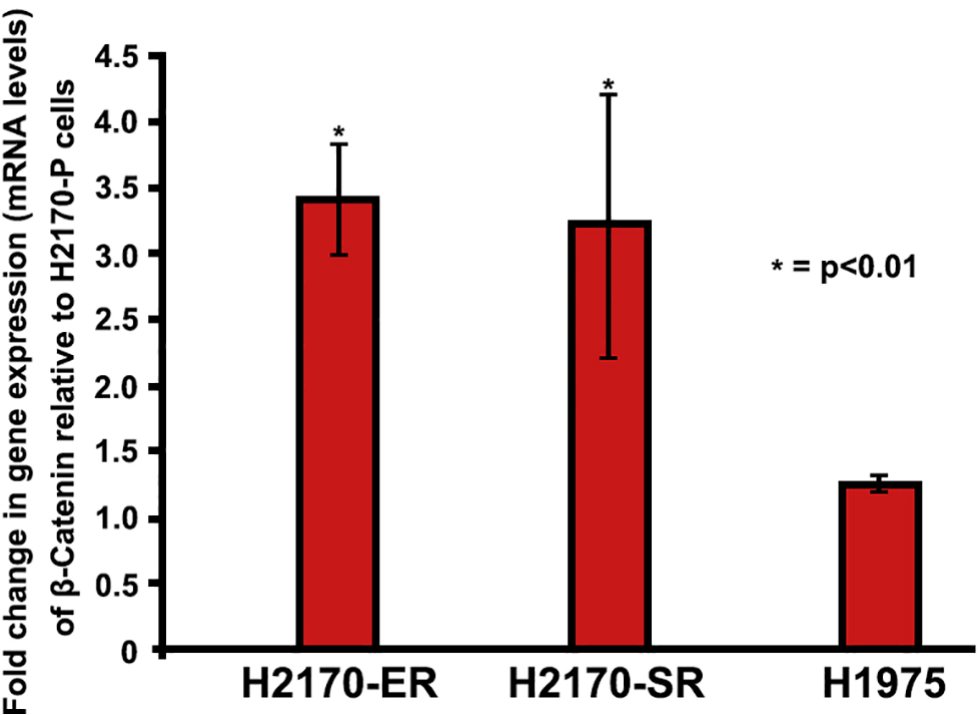
Modulations in gene expression of β-Catenin in H2170 and H1975 cells. H2170 and H1975 cells were plated at 125,000 cells per 35 mm dishes and were allowed to adhere and grow for 24 hours. After which cells were starved (RPMI with 0.5% BSA) for 24 hours and then were processed for RNA collection. Real-Time PCR results show that β-Catenin is upregulated in H2170-ER and H2170-SR cells when compared to H2170-P cells. While, in H1975 (T790M EGFR mutated) cells we did not observe any significant modulation of β-Catenin when compared to H2170-P cells. Expression of each gene was analyzed in triplicate (n = 2, p<0.01).

To further analyze the gene expression of β-Catenin in H2170 cells we performed qPCR experiments as described in the methods section. The results indicate that in H2170-ER cells expression of β-Catenin at mRNA levels was approximately 3.4-fold higher when compared to H2170-P cells (p<0.01). Similarly, the β-Catenin expression in H2170-SR cells was 3.2-fold higher when compared to the H2170-P cells (p<0.01). However, no significant change was observed in β-Catenin gene expression in H1975 cells when compared to H2170-P cells. (Fig 6).

### Effect of Wnt and mtor inhibitors alone and in combination with erlotinib on H1975 cells

**Fig 7:**
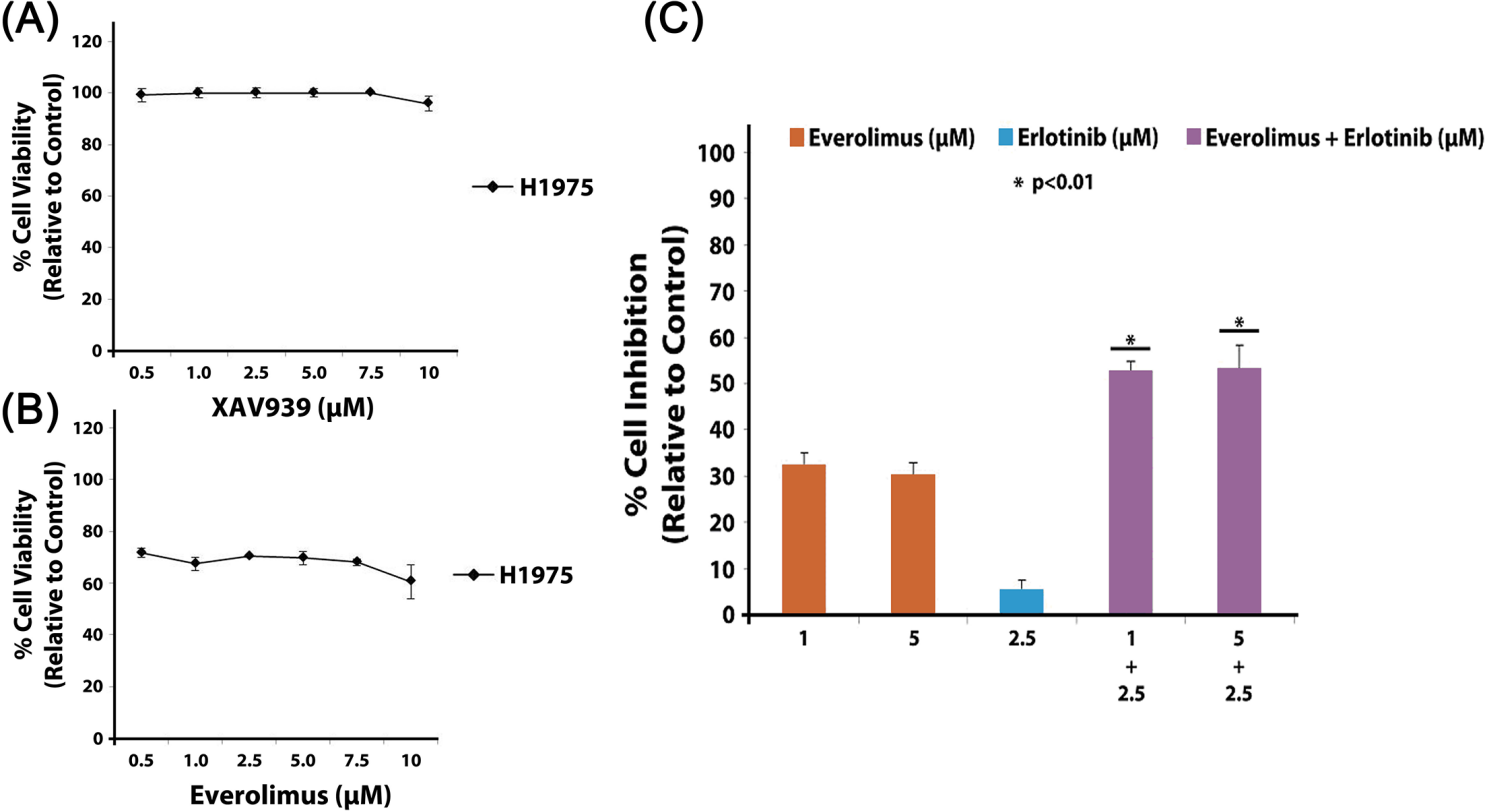
Inhibitory effect of XAV939 and Everolimus on H1975 cells alone and in combination with erlotinib. H1975 cells were plated at 3000 cells per well in a 96 well plate and then treated with varying drug concentrations of XAV939, everolimus and erlotinib alone or in combinations for 72 hours. Cell viability was determined by measuring the absorbance colorimetrically after adding MTT dye. (A) IC_50_ for XAV939 alone in H1975 cells was greater than 10 μM, and similarly (B) the IC_50_ for everolimus alone in H1975 cells was greater than 10 μM. (C) Synergistic inhibition (approximately 53%) of H1975 cells was observed when everolimus and erlotinib were administered in combination. Drug synergism was calculated using Calcusyn v2.0 software. ANOVA analysis was used to determine differences between treatment conditions which were statistically significant (n = 3, p<0.01).

MTT cell viability assays were performed for determining the inhibitory effects of Wnt inhibitor XAV939 and mTOR inhibitor everolimus on H1975 cells. H1975 cell viability was very minimally decreased (<10%) after treatment with XAV939 (up to 10 μM) (Fig 7A), but interestingly, H1975 cell viability was moderately decreased (30 to 40%) after everolimus treatment (up to 10 μM) (Fig 7B). However, selective inhibition of mTOR with everolimus alone was not sufficient to significantly inhibit cell proliferation, so combination therapy with EGFR TKI erlotinib was studied. We observed synergistic effects on inhibition of cell growth in H1975 cells when everolimus and erlotinib were administered in combination at concentrations of 1 μM everolimus with 2.5 μM erlotinib (53% inhibition, p<0.01) and 5 μM everolimus with 2.5 μM erlotinib (54% inhibition, p<0.01) (Fig 7C). The synergistic effect was calculated using Calcusyn software and the CI values were found to be less than 1.

## Discussion

Acquired resistance to EGFR and c-Met TKIs is a common occurrence in patients which limits the overall efficacy of current lung cancer therapies [7]. This study focuses on elucidating key signaling proteins in alternative signaling pathways which result in EGFR/c-Met TKI resistance in lung cancer cell lines which have wild type EGFR or EGFR with T790M mutation. In cells with wild type EGFR, Wnt and mTOR pathways exhibit crosstalk due to proteins which can regulate both of these pathways [8,18,31], such as GSK3β [31,32]. According to the present study, significant modulations of key Wnt and mTOR pathway-related proteins such as active β-catenin, GATA-6 and p-GSK3β confirm the role of these two pathways in H2170-ER and H2170-SR cells. However, in the H1975 cell line which has a T790M mutation, we observed that the mTOR pathway proteins were modulated, but Wnt pathway proteins were not significantly altered. These results suggest that mechanism of resistance may be different in cells with wild type EGFR compared to cells with T790M mutation and different targeting therapies should be used.

This study provides evidence that active β-catenin, which binds to transcription factors TCF/LEF, which are known to transcribe tumorigenesis-enhancing proteins [47,48] is significantly upregulated in both H2170-ER and H2170-SR cells in comparison to H2170-P cells. It has been suggested that over-activation of EGFR enhances nuclear accumulation of active β-catenin [49,50]. In the present study, we observed significantly enhanced nuclear accumulation and increased gene expression of active β-catenin in H2170-ER and H2170-SR cells. As, H2170 cells have upregulated and constitutively activated p-EGFR [17], this suggests that EGFR and Wnt pathways may converge at β-catenin, [51] and thus, cooperatively enhance tumorigenesis in H2170-ER and H2170-SR cells, which has also been suggested by other investigators in other cancers [18,52–55].

The phosphorylated (inactive) form of GSK3β (Ser9), was found to be significantly upregulated in H2170-ER and H2170-SR cells, compared to H2170-P cells, which suggests that it is not able to inhibit the Wnt or mTOR pathways in these cells [31,32,56]. Currently, the role of GSK3β in tumorigenesis and drug resistance is unclear [48]. To our knowledge, this is the first study investigating the role of GSK3β expression in EGFR and c-Met TKI resistant lung cancer cells.

The transcription factor GATA-6 is believed to be involved in the development of cancer by downregulating Wnt antagonist Dickopf-1 [57] and by promoting transcription of important Wnt ligands [37,41,42]. Constitutively activated EGFR in H2170 cells [17], stimulates activation of the RAS/RAF/MEK pathway and its downstream effector ERK [58] which is known to upregulate GATA-6 [39]. Results from this study have shown significant upregulation of GATA-6 in both H2170-ER and H2170-SR cells, compared to H2170-P cells, which further validates the role of GATA-6 in linking EGFR to the Wnt/β-catenin pathway. This is the first study which demonstrates the role of GATA6 in EGFR/c-Met TKI resistance in NSCLC.

Due to the presence of T790M, H1975 cells are highly resistant to EGFR and c-Met TKIs, as seen by higher IC_50_ for erlotinib and SU11274, compared to EGFR wild-type NSCLC cells [17]. In the present study, we observed synergistic inhibitory effects of erlotinib and SU11274 on H1975 cells. This indicates that a combination of a c-Met and EGFR TKI could be used in NSCLC patients with T790M mutation.

Greater than 50% of all acquired resistance to EGFR TKIs is caused by the acquisition of the T790M EGFR mutation [12] and hence, elucidating the mechanism of resistance caused by T790M is important [9]. We observed downregulation of active β-catenin, as well as upregulation of negative Wnt regulator Axin1 in H1975 cells, when compared to H2170-P cells. This suggests that the Wnt/β-catenin pathway may not play a role in TKI resistance in H1975 cells. Interestingly, we observed upregulation of common Wnt co-receptor p-LRP5/6 in H1975 cells, compared to H2170-P cells. Recent studies indicate that this receptor may be involved in the activation of mTOR signaling [59,60]. LRP6 can stimulate the activity of the Akt-mTOR (mTORC1) pathway through an integrated signal with Caveolin-1 [59].

GATA-6, which is known to transcribe important Wnt ligands [39–41], was observed to be significantly downregulated in H1975 cells. Thus, indicating less Wnt ligand is available for binding to LRP5/6 for Wnt/β-catenin pathway activation. Also, the mechanism by which ERK activates GATA-6 is currently unclear, however binding of ERK is required for GATA-6 activation [39]. In H1975 cells ERK may be unable to bind to GATA-6 and hence we observe downregulation of GATA-6 and Wnt/β-catenin. Similarly, we observed significant downregulation of Wnt proteins (active β-catenin, GATA-6, p-LRP5/6 and p-GSK3β) and upregulation of mTOR proteins (p-ERK, p-mTOR and p-p70S6K) in H1975 cells, when compared to H2170-ER and H2170-SR cells.

Based on our results, we suggest that alternative EGFR signaling in H1975 cells (T790M-positive) may be mediated through c-Met and the downstream MAPK/ERK-mTOR pathway. Thus, it may be possible to overcome the resistance through the combination of inhibitors against EGFR, c-Met and mTOR [61,62]. Our results demonstrated that the mTOR inhibitor everolimus or EGFR inhibitor erlotinib alone did not result in significant cell death of H1975 cells. However, we observed significant synergistic inhibition of H1975 cell proliferation after combination treatment of everolimus with erlotinib, supporting the requirement of combinatorial therapies.

In summary, this study demonstrates that in the case of H2170 EGFR and c-Met TKI-resistant NSCLC cells, which are EGFR wild-type, the canonical Wnt and mTOR pathways have prominent roles in facilitating alternative EGFR/c-Met signaling mechanisms, resulting in the development of TKI resistance and cancer cell survival. Thus, it may be possible to break resistance and successfully inhibit cell proliferation and survival in EGFR wild-type cells, through combinatorial inhibition of Wnt and mTOR along with EGFR and c-Met inhibition. However, in the case of EGFR-mutant H1975 NSCLC cells (L858R and T790M positive), our study suggests that alternative EGFR signaling may be occurring mainly through the MAP Kinase and/or PI3K/Akt-stimulated mTOR pathway, resulting in cancer cell proliferation and survival. Thus, in NSCLC patients who have acquired the T790M EGFR TKI resistance-conferring mutation, it may be possible to break resistance, through combining mTOR inhibitors with current EGFR and c-Met TKIs.

## Supporting Information

S1 Fig Expression of total Wnt and mTOR pathway related proteins in H2170 and H1975 cells. H1975 and H2170-P cells were plated at 125,000 cells per dish in 35 mm dishes and starved (RPMI 1640 with 0.5% BSA) for 24 hours before ligand (EGF and HGF) or/and drug (erlotinib and SU11274) treatments and analyzed using western blot. The results were quantified using ImageJ software and no significant modulation of total β-Catenin, total GSK-3β, total ERK and total p70S6K was observed in H1975 cells when compared to H2170-P cells with same treatments. (n = 2).
(TIF)
