# Supplementary figures and images for: Mechanism of Resistance and Novel Targets Mediating Resistance to EGFR and c-Met Tyrosine Kinase Inhibitors in Non-Small Cell Lung Cancer
Source: PLoS One. 2015 Aug 24;10(8):e0136155. doi: 10.1371/journal.pone.0136155 (PMC4547756; doi:10.1371/journal.pone.0136155)

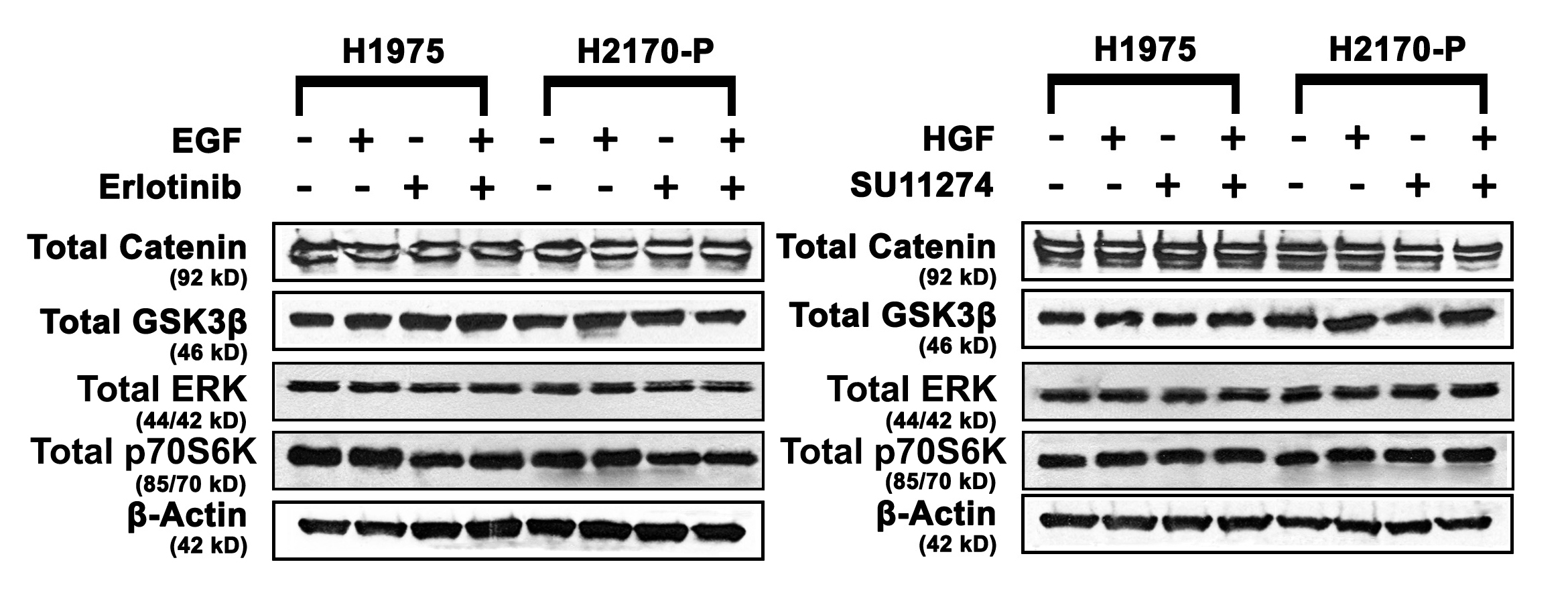

Supplement: S1 Fig — H1975 and H2170-P cells were plated at 125,000 cells per dish in 35 mm dishes and starved (RPMI 1640 with 0.5% BSA) for 24 hours before ligand (EGF and HGF) or/and drug (erlotinib and SU11274) treatments and analyzed using western blot. The results were quantified using ImageJ software and no significant modulation of total β-Catenin, total GSK-3β, total ERK and total p70S6K was observed in H1975 cells when compared to H2170-P cells with same treatments. (n = 2). (TIF) [file pone.0136155.s001.tif]
